# Insights into Living with Kidney Disease

**DOI:** 10.1155/2017/2805684

**Published:** 2017-05-16

**Authors:** Veronica Swallow, Houry Puzantian, Leah Krischock, Ulf Gunnar Bronas

**Affiliations:** ^1^School of Healthcare, University of Leeds, Baines Wing, Leeds LS2 9JT, UK; ^2^School of Nursing, Midwifery, and Indigenous Health, Faculty of Science, Charles Sturt University, Boorooma St, Wagga Wagga, NSW 2650, Australia; ^3^Department of Biobehavioral Health Science, College of Nursing, University of Illinois at Chicago, Chicago, IL, USA; ^4^Sydney Children's Hospital, Randwick, Sydney, NSW, Australia

There are many implications for those across the life-course who are living with kidney disease and their relatives/carers [1, 2]. Chronic kidney disease (CKD) is an important cause of reduced quality of life, morbidity, and death, so early identification is needed to help reduce these dire consequences. Public health approaches to enabling early identification are, therefore, receiving increasing attention. As part of these approaches, self-care is an integral part of daily life for persons across the life-course who are living with kidney disease and often means that patients and/or their carers take responsibility for day-to-day management of complex clinical interventions and treatment regimens, with support (that is often provided remotely) from the health professionals involved in their care [3]. People living with kidney disease can benefit enormously if they receive appropriate support for self-care. However, to understand the best ways to support them we need detailed insights into the challenges of living with kidney disease. The papers presented in this special issue report and discuss current evidence and new innovations; these insights will help professionals who are managing patients living with kidney disease.

In the management of patients receiving hemodialysis therapy, it is notable (as reported by B. El Ghoul et al.) that the etiology of end-stage renal disease is independently associated with arterial stiffness. This is higher among patients who developed renal sequelae of either diabetes mellitus or hypertension as compared with those with a history of either diabetes mellitus or hypertension alone. The clinical implication of this finding can translate into earlier interventions to reduce end-organ complications. Moreover, M. Majernikova et al. note that clinical evaluation and treatment of even mild anemia might reduce the higher risk of mortality in patients with posttransplant anemia in early stages of CKD after kidney transplantation. Furthermore, understanding the cerebro-vascular-renal axis pathophysiological link and its interconnection with the possible protective role of exercise is important for clinicians supporting patients with CKD in order to minimize the risk of loss of independence and improve quality of life (as reported by U. G. Bronas et al.)

Two papers in this issue focus on the psychosocial impact of CKD, in relation to dialysis for adult patients (N. Thomas et al.), and posttransplantation for children and young people (J. Bamford and L. Wirz). Posttransplant psychology annual reviews introduced into one Pediatric Renal Service enabled measurement of psychological distress and quality of life and helped identify those families most likely to benefit from psychological intervention. In N. Thomas et al.'s study, most patients were satisfied with the amount of information they received, although it was recommended that the quality of the information they received could have been improved, in particular concerning the effect of dialysis on individuals' day-to-day life.

Finally, the importance of lobbying policy makers and local water departments to ensure the availability of robust infrastructures support to sustain dialysis, the life-saving therapy for many people living with end-stage renal disease, including at times of natural disasters such as earthquakes, is highlighted by N. Ikegaya et al.

Overall, the new knowledge contained in this special issue makes an important contribution to the literature and can help shape the services and support offered to patients and families living with kidney disease.

*Veronica Swallow* *Veronica Swallow* *Houry Puzantian* *Houry Puzantian* *Leah Krischock* *Leah Krischock* *Ulf Gunnar Bronas* *Ulf Gunnar Bronas*

## References

[1] Jha V., Garcia-Garcia G., Iseki K. (2013). Chronic kidney disease: global dimension and perspectives. *The Lancet*.

[2] Chawla L. S., Bellomo R., Bihorac A. (2017). Acute kidney disease and renal recovery: consensus report of the acute disease quality initiative (ADQI) 16 workgroup. *Nature Reviews Nephrology*.

[3] Ong S. W., Jassal S. V., Porter E., Logan A. G., Miller J. A. (2013). Using an electronic self-management tool to support patients with chronic kidney disease (CKD): a CKD clinic self-care model. *Seminars in Dialysis*.

